# Metformin Modulates Systemic Lipid Remodeling after Traumatic Brain Injury

**DOI:** 10.21203/rs.3.rs-9583079/v1

**Published:** 2026-06-04

**Authors:** Aaron M. Gusdon, Sung-Min Cho, Hua Chen, Farid Radmanesh, Xuefang Ren, Pramod Dash, Jae-Hyek Choi, R. Madelaine Paredes

**Affiliations:** University of Texas Health Science Center; Johns Hopkins University; University of Texas Health Science Center; University of Texas Health Science Center; University of Texas Health Science Center; University of Texas Health Science Center; 59th Medical Wing; 59th Medical Wing

**Keywords:** traumatic brain injury, lipidomics, phospholipids, phosphatidylinositol

## Abstract

**Background:**

Traumatic brain injury (TBI) induces systemic metabolic disturbances, particularly affecting lipid metabolism, which may contribute to secondary injury. Metformin has pleiotropic effects on mitochondrial function and lipid homeostasis, but its impact on the circulating lipidome after TBI remains poorly characterized.

**Methods:**

We performed plasma lipidomic profiling in a porcine TBI model with metformin or control pretreatment. Paired pre- and post-TBI samples from 20 swine enabled within-subject comparisons. Lipidomic data were analyzed using complementary univariate and multivariate approaches, including paired differential abundance testing, principal component analysis (PCA), unsupervised hierarchical clustering with permutation-based cluster purity assessment, and sparse partial least squares discriminant analysis (sPLS-DA).

**Results:**

Unsupervised analyses demonstrated coordinated lipidomic remodeling following TBI, characterized by enrichment of triglycerides and depletion of phosphatidylinositols. Metformin treatment had minimal effects on baseline metabolic profiles, with only subtle multivariate separation and near-chance classification performance. In contrast, post-TBI samples showed clearer treatment-associated differences, including improved separation in PCA, hierarchical clustering, and sPLS-DA models. These differences were driven by coordinated changes across multiple lipid classes rather than large shifts in individual metabolites. Notably, metformin treatment was associated with altered post-TBI patterns of triglycerides containing polyunsaturated fatty acids, phospholipids, lysophospholipids, and cholesteryl esters, suggesting modulation of injury-associated lipid remodeling.

**Conclusions:**

Metformin exerts modest effects on baseline lipid metabolism but is associated with coordinated alterations in the systemic lipidomic response following TBI. These findings support a context-dependent role for metformin as a modulator of post-injury metabolic remodeling and highlight lipid pathways as potential targets for therapeutic intervention after TBI.

## INTRODUCTION

Traumatic brain injury (TBI) triggers profound systemic metabolic disturbances that extend beyond the central nervous system and evolve dynamically over time [1]. In addition to acute neuroinflammatory and neuroenergetic derangements, TBI is accompanied by widespread alterations in peripheral lipid metabolism, reflecting changes in mitochondrial function, membrane remodeling, and inflammatory signaling pathways [2]. These metabolic perturbations have been increasingly recognized as both markers and potential mediators of secondary injury processes, including cerebral edema and vascular dysfunction [3]. Despite lipid metabolites being associated with outcomes after TBI [4, 5], treatments aimed at augmenting phospholipid synthesis have not demonstrated improved outcomes [6]. Therefore, new approaches to understand and modify the circulating lipid biomarker signature after TBI are needed. In this study, we specifically modeled moderate TBI, as it represents a diagnostically challenging clinical window between overt structural damage (severe TBI) and subtle, often delayed concussive signs (mild TBI). This middle-ground severity is highly relevant to both clinical and operational decision-making where immediate triage is critical

Metformin has attracted growing interest for its pleiotropic effects on energy metabolism, mitochondrial function, and lipid homeostasis. Widely prescribed for the treatment of type 2 diabetes, metformin activates AMP-activated protein kinase (AMPK), inhibits mitochondrial complex I, and modulates fatty acid synthesis and oxidation pathways [7–9]. Beyond glycemic control, metformin has been shown to exert anti-inflammatory, vasculoprotective, and neuroprotective effects in both experimental and clinical settings [10–12]. Preclinical studies suggest that metformin can influence outcomes after neurological injury, including ischemic stroke and TBI, potentially through modulation of oxidative stress, microglial activation, and systemic metabolic responses [13–15]; however, it is unknown whether metformin meaningfully altered systemic lipid metabolism after TBI.

Prior metabolomic studies in TBI and related critical illness contexts have consistently identified lipid pathways, including triglycerides, phospholipids, lysophospholipids, and sphingolipids as key components of the injury response [16, 17]. These lipid classes are closely linked to membrane integrity, mitochondrial energetics, and inflammatory signaling, making them biologically plausible mediators of secondary brain injury. However, existing studies have largely relied on rodent models, which incompletely recapitulate the metabolic and physiological complexity of human TBI, or on clinical cohorts in which pre-injury samples are unavailable, precluding paired analyses that account for inter-individual metabolic variability. Importantly, the gyrencephalic organization and high white-matter–to–gray-matter ratio of the porcine brain more closely reflect human neuroanatomy than lissencephalic rodent models, strengthening its translational relevance for TBI-related metabolic studies [18, 19].

In the present study, we applied complementary unsupervised and supervised metabolomic analyses to investigate the effects of metformin treatment on systemic lipid metabolism before and after TBI in a large animal (porcine) model. This design enabled paired, within-subject comparisons across injury states while preserving clinically relevant physiology. We hypothesized that metformin would exert minimal effects on baseline metabolic profiles but would modify lipid remodeling following TBI. By integrating multiple bioinformatic approaches, we aimed to determine whether metformin influences metabolic state prior to injury or acts primarily as a modulator of the injury-associated metabolic response.

## METHODS

### Animal Models and Experimental Design

The protocol was approved by the United States Air Force, 59th Medical Wing (MDW) Institutional Animal Care and Use Committee (IACUC) and conducted in accordance with the regulations and guidelines of the Animal Welfare Act, the National Research Council Guide for the Care and Use of Laboratory Animals, and the American Association for the Accreditation of Laboratory Animal Care (AAALAC). All procedures in the study were conducted at the Clinical Investigations and Research Support (CIRS) Laboratory. The ARRIVE guidelines were used to ensure proper reporting of methods, results, and discussion. The study was funded by the Department of Defense DHA Restoral.

Twenty male Yorkshire swine (weighing 35–45 kg) were utilized in this study. Following a seven-day acclimation period, the animals were randomized (1:1) to receive either a standard diet (Control; n = 10) or a diet top-dressed with metformin (n = 10; 850 mg twice daily) for seven days prior to TBI induction.

### Surgical Procedures and TBI Induction

Prior to surgery, all animals were fasted for 16 hours. On the day of the procedure, swine were intubated and maintained under anesthesia by personnel from the Clinical Investigation and Research Support (CIRS) of 59th Medical Wing (Lackland Air Force Base, TX), with continuous physiological monitoring to ensure the absence of undue distress. TBI was induced using a blunt impact model developed at the 59th MDW and similar to the one described by Earle *et al*[20]. A controlled impact was delivered via a powder-actuated piston striking an aluminum disk positioned on the animal’s cranial vault to ensure uniform force distribution. Animals were continuously monitored for 6 hours following TBI.

Blood samples were collected prior to humane euthanasia in serum blood collection tubes (gold top vacutainers) immediately prior to TBI and after the six-hour observation period. Following collection, blood samples were allowed to sit at room temperature for 20 minutes and then centrifuged at 3,000 rpm for 10 minutes at 4°C for serum separation. The resulting serum was then aliquoted and stored at −80°C until the time of lipidomic analysis. All 20 swine had serum samples available prior to TBI, however a serum sample was not able to be collected from one swine after TBI.

### Lipidomics and data preprocessing

Serum lipidomic profiling was performed at the Metabolomics Facility at The University of Texas MD Anderson Cancer Center using established mass spectrometry–based lipidomics workflows. Briefly, serum samples were processed using standardized organic solvent extraction protocols optimized for broad lipid class coverage, followed by high-resolution liquid chromatography–mass spectrometry (LC–MS) analysis. Lipid species were identified and quantified based on accurate mass, retention time, and comparison with internal standards and curated lipid libraries, with annotation at the level of lipid class and acyl chain composition where applicable. Raw peak intensities were subjected to facility-standard quality control procedures, including assessment of internal standard performance, evaluation of technical reproducibility, and removal of low-abundance or poorly detected features. Following initial processing, normalized metabolite abundance data were returned for downstream statistical analysis.

All subsequent analyses were conducted in R (version 4.4.2). Metabolite intensities were log_2_-transformed to reduce right-skewness and stabilize variance, then standardized by z-score normalization across metabolites to ensure equal weighting of features in multivariate analyses. Metabolites were annotated by lipid class for downstream class-level summaries and visualization. Metabolites with > 10% missingness were excluded. These preprocessing steps were applied uniformly across all analyses to facilitate comparability between unsupervised clustering, principal component analysis, supervised multivariate modeling, and univariate differential abundance testing.

### Differential abundance analysis

Differential abundance between pre- and post-TBI samples was assessed on a per-metabolite basis using a paired analytical framework. For each metabolite, log_2_ fold changes were calculated as post-TBI relative to pre-TBI within matched samples. Statistical significance was evaluated using paired Wilcoxon signed-rank tests, with correction for multiple comparisons applied across metabolites and false discovery rate (FDR)–adjusted q-values reported. Results were visualized using volcano plots, with metabolites colored by lipid class. Thresholds for signficiance included |log_2_ fold change| > 1 and q < 0.05.

### Principal Component Analysis (PCA)

Principal component analysis (PCA) was performed to assess global metabolomic differences between pre- and post–traumatic brain injury (TBI) samples. PCA was conducted using singular value decomposition as implemented in the prcomp() function in R, with centering enabled and no additional scaling beyond prior z-score normalization. Scores for the first two principal components (PC1 and PC2) were extracted and visualized, with samples colored by experimental group. The proportion of variance explained by each component was calculated from the corresponding eigenvalues and reported on the plot axes.

To identify metabolites contributing most strongly to the observed separation, loadings from PC1 were extracted from the same PCA model. Metabolites were ranked by the absolute value of their PC1 loadings, and the top contributors were visualized in bar plots. Loadings were annotated by lipid class to facilitate biological interpretation and to link unsupervised multivariate structure with downstream differential abundance analyses.

### Sparse Partial Least Squares Discriminant Analysis (sPLS-DA)

Sparse partial least squares discriminant analysis (sPLS-DA) was performed using the mixOmics R package[21, 22] to evaluate whether metformin pretreatment was associated with differences in pre- and post-TBI metabolomic profiles. Normalized metabolite abundances were analyzed with metabolites treated as features and samples as observations; data were transposed as required by mixOmics input specifications. No additional scaling was applied beyond upstream normalization.

Models were constructed using two latent components. Feature selection was incorporated via sparsity constraints, with the number of metabolites retained per component (keepX) optimized using repeated five-fold cross-validation (50 repeats). Candidate keepX values ranging from 5 to 100 metabolites per component were evaluated. Model performance was assessed using the balanced error rate (BER) with maximum distance classification, and the keepX configuration minimizing cross-validated BER was selected for the final model. Sample separation was visualized using sPLS-DA score plots, and metabolites contributing to class separation were identified from component loadings.

### Lipid Class–Level Summary Analysis

To assess coordinated changes at the lipid class level, metabolites were grouped according to annotated lipid class. For each class, the median log_2_ fold change (post-TBI/pre-TBI) was calculated, along with interquartile ranges to summarize within-class variability. Results were visualized as lipid class–level effect size plots, with a reference line at zero indicating no change. Lipid class color schemes were applied consistently across all figures to facilitate cross-panel comparison.

### Unsupervised Clustering and Cluster Purity Assessment

Unsupervised hierarchical clustering was performed on scaled metabolite abundance data to identify patterns of similarity across samples without incorporating clinical labels. Cluster purity was calculated as the proportion of samples within each cluster belonging to the dominant clinical group. Statistical significance of observed cluster purity was assessed using permutation testing with 1,000 random label shuffles. Cohort-specific cluster purity was assessed descriptively and evaluated using permutation testing within each cohort, recognizing reduced statistical power due to stratified analyses.

## RESULTS

### Lipidomics data

A total of 617 lipid metabolites were included in analyses. Classes of lipids included neutral lipids [triglycerides (TGs) and diacylglycerides (DGs)], phospholipids [phosphatidylcholines (PCs), phosphatidylethanolamines (PEs), phosphatidylserines (PSs), phosphatidylinositols (PIs)], lysophospholipids [lysophosphatidylcholines (LPCs), lysophosphatidylethanolamines (LPEs)], sphingolipids [sphingomyelins (SMs), ceramides (Cers), hexosylceramides (Hex)], acylcarnitines (AcCa), and cholesterol and cholesterol esters [free cholesterol, cholesterol esters (CEs)].

### Global metabolic differences before and after TBI

**Figure 1:**
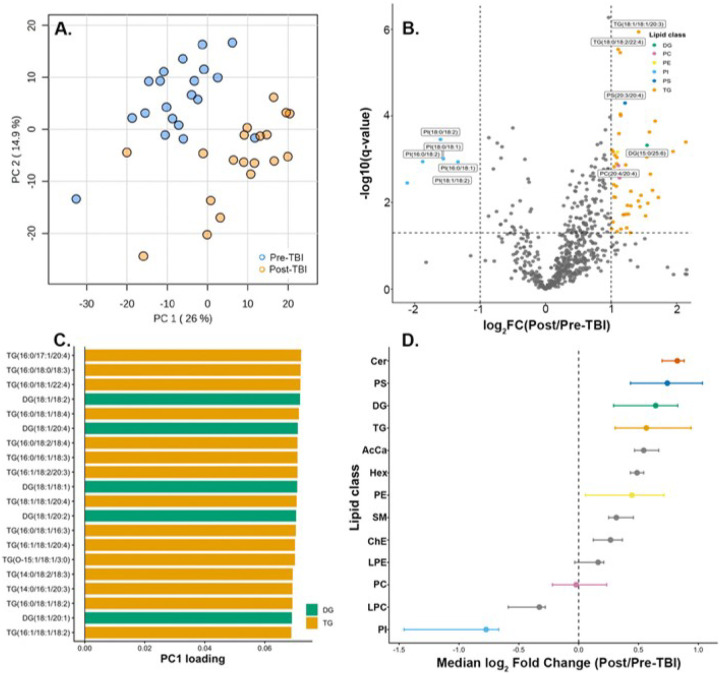
Lipidomic changes following TBI. (A) PCA of normalized plasma lipidomic profiles demonstrating separation between pre-TBI and post-TBI samples. Percent variance explained by PC1 and PC2 is indicated on the axes. (B) Volcano plot showing differential lipid abundance after TBI, with log_2_ fold change (post/pre-TBI) plotted against −log_10_(q-value). Lipids significantly increased (log_2_FC > 1, q < 0.05) or decreased (log_2_FC < −1, q < 0.05) are highlighted and colored by lipid class; selected top 5 increased and top 5 decreased are annotated. (C) Bar plot of the top metabolites contributing to separation along PC1, ranked by absolute PC1 loading. Bars are colored by lipid class, highlighting dominant contributions from triglycerides (TG) and diglycerides (DG). (D) Lipid class–level summary of post-TBI changes, showing median log_2_ fold change (post/pre-TBI) for each lipid class with error bars representing interquartile ranges. The dashed vertical line indicates no change (log_2_FC = 0). *Abbreviations*: TBI, traumatic brain injury; PCA, principal component analysis; PC1/PC2, principal components 1 and 2; FC, fold change; q-value, false discovery rate–adjusted p value; TG, triglyceride; DG, diglyceride; PC, phosphatidylcholine; PE, phosphatidylethanolamine; PI, phosphatidylinositol; PS, phosphatidylserine; LPC, lysophosphatidylcholine; LPE, lysophosphatidylethanolamine; SM, sphingomyelin; Cer, ceramide; ChE, cholesteryl ester; AcCa, acylcarnitine; Hex, hexosylceramide.

Unsupervised principal component analysis (PCA) of normalized plasma lipidomic profiles demonstrated partial separation between pre- and post-TBI samples (Fig. 1A). The first principal component (PC1), accounting for 26% of the total variance, largely distinguished post from pre-TBI samples, while PC2 explained an additional 14.9% of variance and reflected within-group heterogeneity. Although overlap between groups was present, the observed separation along PC1 suggests a coordinated shift in the circulating lipidome associated with TBI rather than complete global divergence.

Volcano plot analysis comparing pre- and post-TBI samples identified a class-specific pattern of differential metabolite abundance (Fig. 1B). TG species were predominantly enriched post-TBI, with multiple TGs exhibiting large positive log_2_ fold changes and statistically significant q-values. In contrast, several PI species demonstrated significant negative log_2_ fold changes, indicating relative depletion after TBI. Other lipid classes, including PSs, PEs, and DGs, showed more modest and heterogeneous changes. A full list of significantly altered metabolites is shown in Table S1.

To identify metabolites driving the observed separation in PCA, we examined PC1 loadings from the same analysis (Fig. 1C). Metabolites with the largest positive PC1 loadings—corresponding to higher abundance post-TBI—were overwhelmingly enriched for TG species, with additional contributions from DGs. These findings indicate that accumulation of neutral lipid species is a major determinant of the TBI-associated metabolic shift captured by PC1, directly linking the unsupervised PCA structure to the differential abundance results.

Consistent with both the volcano plot and PCA loading analysis, class-level aggregation of metabolite changes revealed coordinated lipid remodeling across major lipid classes (Fig. 1D). TGs, Cers, PSs, DGs, Hex, and AcCas exhibited positive median log_2_ fold changes. Conversely, PI species and LPCs showed negative median log_2_ fold changes, indicating opposing class-level trends. Other lipid classes demonstrated smaller effect sizes centered closer to zero. Together, these results support a model in which TBI is associated with broad TG enrichment accompanied by relative depletion of PI and LPC species, rather than isolated changes in individual metabolites.

### Hierarchical clustering

**Figure 2:**
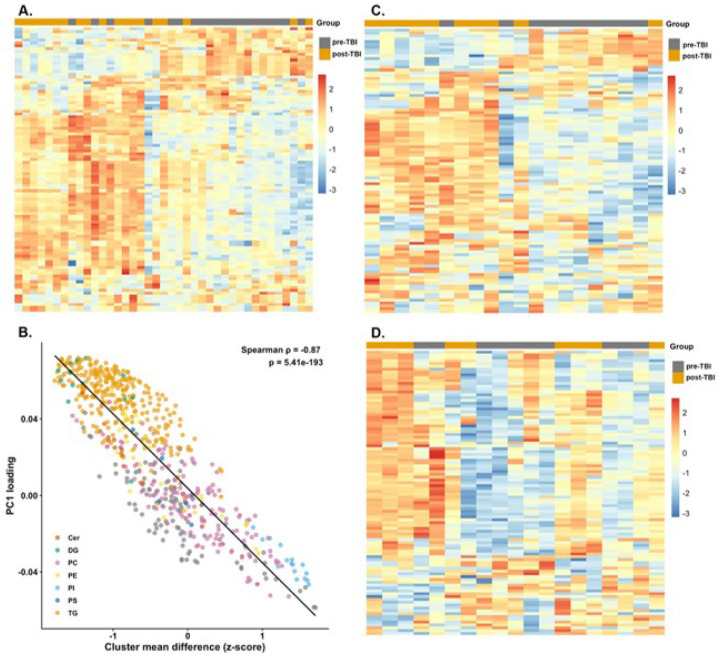
Lipid class–level structure underlying TBI-associated metabolomic changes. (A) Unsupervised hierarchical clustering heatmap of scaled plasma lipid abundances across all samples. Rows represent individual lipid species and columns represent samples, with color indicating z-score–normalized abundance. The annotation bar indicates sample group (pre- vs post-TBI). (B) Relationship between lipid-specific contribution to global variance and group-level abundance differences. Scatter plot showing PC1 loadings from PCA versus cluster mean difference (z-score) between pre- and post-TBI samples for each lipid species. Points are colored by lipid class. The fitted regression line is shown, with Spearman correlation coefficient (ρ) and associated p value indicated, demonstrating a strong inverse relationship between PC1 contribution and group-level abundance shifts. (C) Unsupervised hierarchical clustering heatmap of scaled plasma lipid abundances across control samples. (D) Unsupervised hierarchical clustering heatmap of scaled plasma lipid abundances across metformin treated samples. *Abbreviations*: PCA, principal component analysis; PC1, principal component 1; Cer, ceramide; DG, diglyceride; PC, phosphatidylcholine; PE, phosphatidylethanolamine; PI, phosphatidylinositol; PS, phosphatidylserine; TG, triglyceride.

Unsupervised hierarchical clustering of the most variable metabolites yielded two primary sample clusters that were enriched for pre- and post-TBI samples, respectively (Fig. 2A). Cluster purity analysis demonstrated that 79% of samples within each cluster belonged to the same group (pre- and post-TBI), and this degree of concordance was significantly greater than expected by chance (permutation p = 0.001), supporting the presence of coordinated metabolic differences between pre- and post-TBI samples.

To assess whether unsupervised sample clustering and principal component analysis captured shared underlying metabolic structure, cluster-defining metabolites were compared with PC1 loadings from the same PCA model. Metabolites exhibiting the largest mean differences between clusters also demonstrated the largest absolute PC1 loadings, with a strong monotonic relationship between cluster effect size and PC1 contribution (Fig. 2B, Spearman ρ = −0.87, p < 0.001). TG species were predominantly enriched among metabolites with positive cluster differences and positive PC1 loadings, whereas PI species showed opposing trends. These findings indicate that the metabolic features driving unsupervised sample clustering are the same features underlying separation along PC1, supporting a coherent lipid-driven structure across independent unsupervised analyses.

### Effect of metformin treatment on lipid metabolites

Volcano plot analysis using conservative criteria (false discovery rate–adjusted p < 0.05 and fold change > 2 or < 0.5) did not identify any individual lipid metabolites reaching statistical significance when comparing metformin treated or untreated groups prior to or after TBI. This suggested that lipidomic differences between groups were subtle and distributed across multiple correlated features rather than driven by large changes in single lipids. Multivariate modeling was therefore used to identify coordinated metabolic patterns. To capture potential multivariate structure and interactions among metabolites, sPLS-DA was applied. sPLS-DA is well suited for high-dimensional metabolomic data, as it identifies combinations of correlated metabolites that jointly discriminate groups while applying feature selection to limit overfitting. This approach revealed structured lipid signatures that were not detectable by univariate testing alone, highlighting coordinated alterations in lipid metabolism rather than isolated metabolite changes.

**Figure 3:**
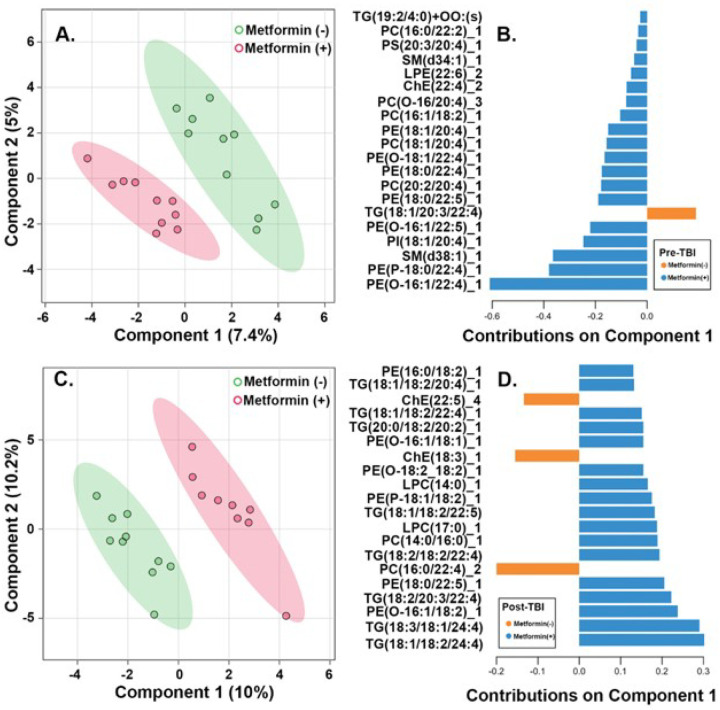
Metformin-associated modulation of lipidomic responses to TBI. (A) sPLS-DA scores plot of pre-TBI plasma lipidomic profiles stratified by metformin treatment status, demonstrating limited separation between metformin-treated and untreated groups. Shaded ellipses represent 95% confidence intervals. (B) Loadings on component 1 from the baseline sPLS-DA model, highlighting lipid species contributing to group separation prior to TBI. Bars are colored by metformin treatment group. (C) sPLS-DA scores plot of post-TBI plasma lipidomic profiles, demonstrating improved separation between metformin-treated and untreated groups relative to baseline. Percent variance explained by each component is indicated on the axes. (D) Loadings on component 1 from the post-TBI sPL-DA model, highlighting coordinated contributions from multiple lipid classes to post-TBI group separation. *Abbreviations*: sPLS-DA, sparse partial least squares discriminant analysis; PC, phosphatidylcholine; PE, phosphatidylethanolamine; PI, phosphatidylinositol; PS, phosphatidylserine; TG, triglyceride; DG, diglyceride; LPC, lysophosphatidylcholine; LPE, lysophosphatidylethanolamine; SM, sphingomyelin; Cer, ceramide; ChE, cholesteryl ester.

To evaluate whether metformin treatment was associated with a distinct baseline metabolic phenotype prior to TBI, sPLS-DA was performed restricted to baseline samples, comparing metformin-treated and control groups. The sPLS-DA scores plot demonstrated partial separation between groups along the first component (Fig. 3A); however, this separation was not sufficient to yield robust classification. On the first component, the model selected only five metabolites as contributing features (Fig. 3B, **Table S2**; top 20 metabolites included by absolute loading on component 1 for visualization only), yet cross-validated overall and balanced error rates (BER) remained near chance (0.56). Inclusion of a second component did not improve model performance (BER 0.54), indicating that the observed separation reflects a modest directional shift rather than a stable discriminative signature. Together, these findings suggest that while metformin treatment is associated with subtle baseline metabolic differences, these differences are not sufficiently consistent to reliably distinguish groups prior to injury.

In contrast to baseline analyses, post-TBI samples demonstrated clearer separation between treatment groups in the sPLS-DA scores plot (Fig. 3C), particularly with inclusion of a second component. Model performance improved with additional dimensionality, with cross-validated overall and BER decreasing from approximately 0.47 on component 1 to 0.38 on component 2. Tuning identified a relatively large number of contributing metabolites on each component (keepX = 100), indicating that treatment-associated differences post-TBI reflect coordinated changes across multiple metabolic features rather than a sparse signature. Loadings on the first component were enriched for glycerolipids and phospholipids, including multiple TG and ether-linked phospholipid species (Fig. 3D, demonstrating top 20 metabolites; all metabolites for both components are found in **Table S3**).

**Figure 4:**
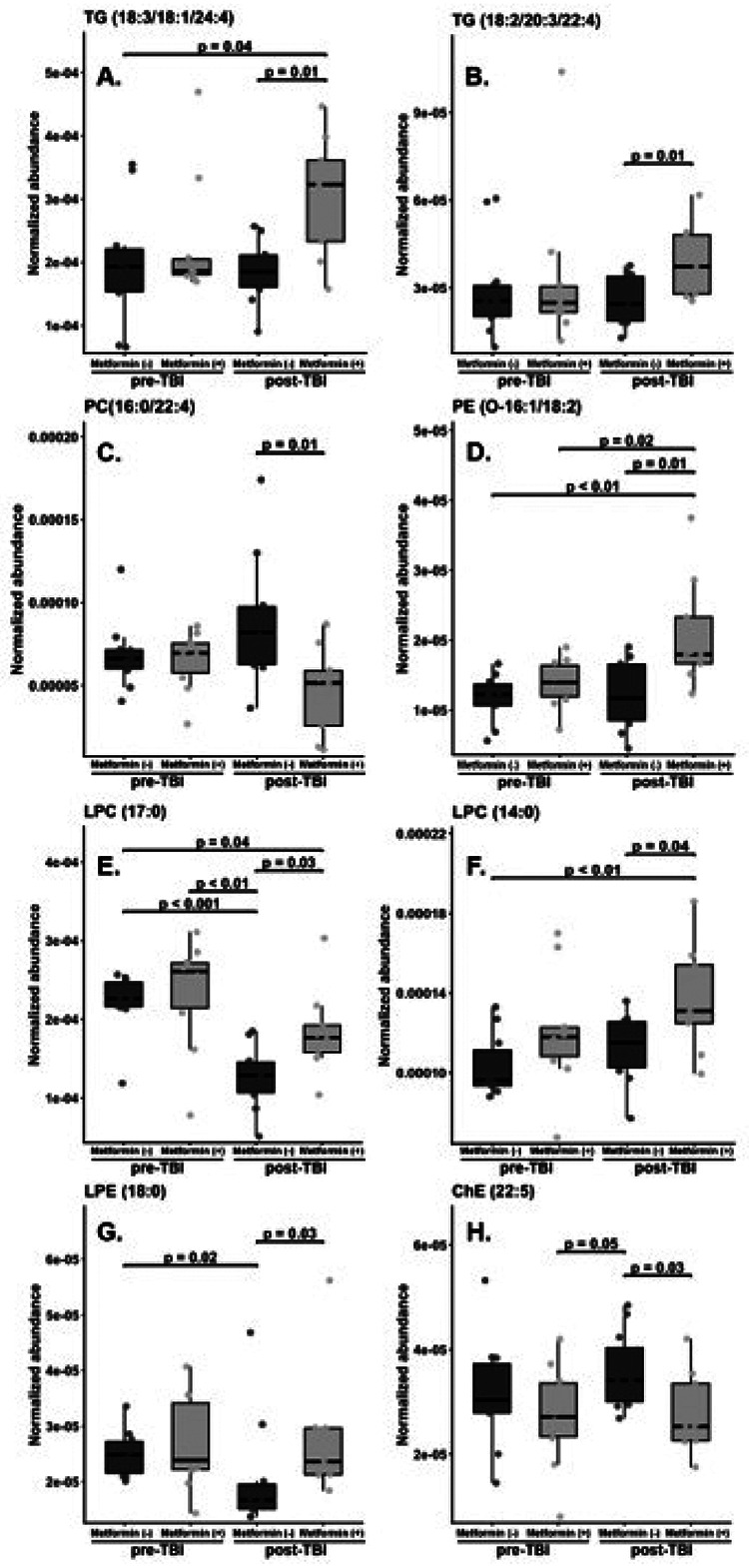
Univariate analysis of representative lipid species identified by sPLS-DA. (A–G) Boxplots showing normalized abundances of selected lipid species from multiple lipid classes that contributed to group separation in the post-TBI sPLS-DA model. Individual points represent individual samples. Group comparisons include pre- and post-TBI samples stratified by metformin treatment status. Horizontal bars indicate pairwise comparisons, with corresponding *p* values shown. *Abbreviations*: TG, triglyceride; PC, phosphatidylcholine; PE, phosphatidylethanolamine; LPC, lysophosphatidylcholine; LPE, lysophosphatidylethanolamine; ChE, cholesteryl ester.

To further interrogate lipid classes contributing to treatment-associated separation after TBI, representative metabolites from the classes identified as major contributors in the sPLS-DA loadings were examined. As shown in Fig. 4, multiple TG, phosphatidylcholine (PC), phosphatidylethanolamine (PE), lysophospholipid (LPL), and cholesterol ester (ChE) species demonstrated significant differences according to metformin treatment, with more prominent changes noted after TBI. In particular, although TGs in general were elevated after TBI, TGs consisting of polyunsaturated free fatty acids (PUFAs) were enriched with metformin treatment after TBI (Fig. 4A,B). PCs were decreased with metformin treatment (Fig. 4C), whereas PEs were increased (Fig. 4D). LPLs, such as LPCs (Fig. 4E,F) and lysophosphatidylethanolamines (LPE) (Fig. 4G), were generally decreased after TBI with metformin treatment increasing their levels. Conversely, ChE were decreased with metformin treatment (Fig. 4H). These findings provide metabolite-level validation of the multivariate patterns observed in the supervised analysis and indicate that treatment-associated differences after TBI reflect coordinated changes across multiple lipid classes.

## DISCUSSION

In this study, we combined unsupervised and supervised metabolomic analyses to characterize the effects of metformin treatment on systemic lipid metabolism both before and after TBI. Across multiple complementary approaches, we identified distinct patterns pre- and post-TBI, indicating that metformin exerts context-dependent effects on lipid metabolism. Using paired analysis before and after injury, we showed that TBI causes broad increases in lipids such as TGs and DGs but decreases in PIs and LPCs. Prior to injury, baseline metabolomic profiles demonstrated only subtle differences between metformin-treated and untreated groups, with partial multivariate separation but limited classification performance, suggesting minimal pre-existing metabolic reprogramming. In contrast, following TBI, metformin treatment was associated with more pronounced and coordinated alterations in lipid metabolism. These post-injury differences were driven by distributed contributions across multiple lipid classes, including PUFA containing TGs, LPLs, PC/PE, and ChE. Together, these findings indicate that metformin substantially modulates the lipid remodeling that accompanies the metabolic response to TBI.

Across paired pre- and post-TBI samples, we observed a consistent pattern of systemic lipid remodeling characterized by enrichment of neutral lipids, TGs and DGs, alongside relative depletion of membrane-associated phospholipids, including LPCs and PIs. This pattern is consistent with prior plasma and serum metabolomic studies in both experimental and clinical TBI, which have identified glycerolipid accumulation and disruption of choline-containing phospholipids as core features of the acute injury response [2, 3, 5, 16]. Elevations in circulating TGs and DGs have been observed in various critical illnesses and likely occur due to stress-induced alterations in hepatic lipid packaging, catecholamine-mediated lipolysis, and impaired peripheral fatty-acid utilization, reflecting a systemic shift from energy storage toward redistribution after acute injury [23–25]. Conversely, reductions in LPCs and PIs are biologically plausible given their central roles in membrane turnover, phospholipid remodeling, and inflammatory signaling, processes that are rapidly engaged following brain injury [26–28]. Depletion of LPCs has been associated with greater injury severity and unfavorable neurological outcomes in human traumatic brain injury cohorts [4, 5], supporting their role as sensitive indicators of membrane disruption and systemic metabolic stress. In parallel, reductions in PIs likely reflect increased utilization of phosphoinositide pools required for membrane repair, vesicular trafficking, and intracellular signaling during conditions of acute cellular stress following brain injury [27]. Together, these findings support a model in which early post-TBI systemic metabolism favors neutral lipid accumulation at the expense of membrane-associated phospholipids, consistent with coordinated lipidome remodeling rather than isolated changes in individual metabolites.

Multivariate modeling and targeted univariate analyses indicate that metformin-associated metabolic differences after TBI are driven by coordinated alterations across multiple lipid classes rather than isolated metabolite changes. In contrast, metformin exerted minimal effects on baseline lipidomic profiles prior to injury, suggesting that its metabolic impact is largely contingent on the post-injury physiological state. Following TBI, metformin treatment was associated with remodeling of lipid pathways implicated in energy storage, membrane turnover, and inflammatory signaling, including TGs, phospholipids, LPLs, and ChEs. The preferential emergence of metformin-associated metabolic differences after TBI likely reflects the drug’s dependence on underlying energetic and inflammatory stress to exert measurable effects[29–31]. Whereas baseline metabolic homeostasis limits the measurable impact of metformin on circulating lipid profiles, the profound mitochondrial dysfunction, inflammatory activation, and lipid overflow induced by TBI create a metabolic milieu in which metformin can redirect lipid handling across multiple interconnected pathways [14, 32, 33].

The pattern of lipid remodeling observed with metformin treatment after TBI is biologically consistent with a shift toward membrane repair, anti-inflammatory signaling, and improved cellular resilience. The relative increase in PEs alongside reductions in PCs suggests altered phospholipid remodeling favoring membrane curvature, fusion, and autophagic processes, which are critical for cellular repair following injury. PEs play a central role in mitochondrial membrane integrity and autophagosome formation [34, 35], whereas excessive PC abundance has been linked to rigid membrane structures that may impair dynamic remodeling [36]. Restoration or preservation of PE pools after TBI may therefore support mitochondrial function and cellular recovery.

Metformin-associated increases in LPLs, including LPCs and LPEs, further support a reparative lipidomic profile. Although LPC depletion has been associated with injury severity and unfavorable outcomes after TBI[4, 5], controlled restoration of LPLs pools may facilitate membrane turnover and phospholipid reacylation through Lands’ cycle, promoting repair rather than inflammation[37]. Importantly, this effect appears context dependent, as excessive or unregulated lysophospholipid signaling can be proinflammatory, whereas balanced availability supports membrane homeostasis[38]. Finally, reductions in ChEs with metformin treatment may reflect decreased lipid droplet accumulation and improved cholesterol trafficking, processes linked to reduced neuroinflammation and enhanced synaptic recovery[39, 40]. Together, these coordinated changes suggest that metformin shifts post-TBI lipid metabolism away from maladaptive lipid accumulation and toward a state that supports membrane repair, mitochondrial function, and attenuated inflammatory signaling.

Several limitations of this study warrant consideration. First, although paired sampling and multivariate modeling strengthens inference regarding injury-associated metabolic changes, the present analyses are exploratory and not designed to establish causality between metformin exposure and lipidomic remodeling. As such, the observed associations should be interpreted as hypothesis-generating rather than definitive evidence of a direct mechanistic effect. Second, while the porcine TBI model provides important physiological and metabolic relevance compared with rodent systems, it does not fully recapitulate the heterogeneity of human TBI, including variability in injury mechanisms, comorbidities, and clinical management. Third, although multivariate approaches revealed coordinated, class-level lipid changes, classification performance was modest and individual metabolites rarely achieved strong univariate significance, reflecting distributed pathway-level effects and limited statistical power to detect small effect sizes. Finally, metformin dosing and timing were fixed in this study, precluding assessment of dose-response relationships or temporal dynamics of metabolic remodeling. Despite these limitations, the differential clustering patterns observed between treated and untreated groups support the hypothesis that metformin exposure influences the systemic metabolic phenotype in the post-injury state and provide a rationale for future mechanistic and translational studies.

## CONCLUSIONS

In summary, this study demonstrates that TBI is associated with coordinated, class-level remodeling of the circulating lipidome and that metformin modifies this metabolic response in a context-dependent manner. Metformin exerted minimal effects on baseline lipid profiles but was associated with distinct alterations in post-TBI lipid remodeling, characterized by coordinated changes across multiple lipid classes rather than isolated metabolite shifts. These findings suggest that metformin primarily influences the systemic metabolic response to brain injury rather than the pre-injury metabolic state, highlighting lipid pathways as potential targets for therapeutic modulation after TBI. Future studies are needed to define the mechanistic basis of these effects and to determine whether metformin-associated metabolic remodeling translates into improved neurological outcomes. In particular, integrating comprehensive behavioral assessments and longer-term treatment paradigms will be critical for establishing causal links between metformin exposure, sustained metabolic reprogramming, and functional recovery after brain injury.

## Supplementary Material

Supplementary Files

This is a list of supplementary files associated with this preprint. Click to download.

- SupplementaryMaterialTSR.docx

## Data Availability

All data will be made available upon reasonable request to the corresponding author.
